# The possible involvement of oxidative stress in the oocyte ageing process in goldfish *Carassius auratus* (Linnaeus, 1758)

**DOI:** 10.1038/s41598-019-46895-1

**Published:** 2019-07-18

**Authors:** Azadeh Mohagheghi Samarin, Azin Mohagheghi Samarin, Tone-Kari Knutsdatter Østbye, Bente Ruyter, Sabine Sampels, Viktoriia Burkina, Miroslav Blecha, Tomas Policar

**Affiliations:** 1 University of South Bohemia in Ceske Budejovice, Faculty of Fisheries and Protection of Waters, South Bohemian Research Center of Aquaculture and Biodiversity of Hydrocenoses, Research Institute of Fish Culture and Hydrobiology, Zátiší 728/II, 389 25, Vodňany, Czech Republic; 2 Nofima (Norwegian Institute of Food, Fisheries and Aquaculture Research), P.O. Box 210, NO-1431, Ås, Norway; 3 Swedish University of Agricultural Sciences, Department of Molecular Sciences, PO Box 7015, 75007 Uppsala, Sweden

**Keywords:** Reproductive biology, Ageing, Animal physiology, Transcriptomics

## Abstract

Decreasing egg quality following oocyte ageing is a major restricting factor for the breeding programs. The mechanisms behind this process has not yet been clarified. To examine the possible involvement of oxidative stress in the oocyte ageing process, the relative mRNA abundance of specific transcripts were determined in oocytes collected from 6 females and incubated *in vitro* for 18 hours post stripping at 20 °C in goldfish *Carassius auratus*. During the 18 hour-post-stripping ageing of the oocytes, relative mRNA levels of candidate transcripts involved in oxidative injury, mitochondrial function and stress response, cell cycles, apoptosis, reproduction and germ line speciation and developmental competence were measured by real-time PCR. None of the relative mRNA abundance of the examined genes were significantly altered through oocyte ageing. In addition, the amount of thiobarbituric acid reactive substances (TBARS), an indicator of lipid peroxidation, did not change over time following stripping. The activity of the antioxidant enzymes also remained constant during oocyte ageing. The results of the current study indicated that oxidative stress unlikely plays a role as an initiator or promotor in the progress of oocyte ageing in goldfish.

## Introduction

The meiosis in female germ cell is accompanied by changes in nucleus and cytoplasm, finally preparing oocyte to be fertilized and subsequently develop into an embryo^1^. It is known that ovulated oocytes could have successful fertilization when the fusion of sperm and ovum takes place in the optimal period for oocyte fertilization. If post-ovulatory oocytes have prolonged residence in the oviduct or body cavity (*in vivo*) or in the culture media (*in vitro*), the ageing of oocyte occur which significantly reduces the oocyte quality and may cause abnormal development of arising embryos^1^. Oocytes more than the optimal period for fertilization after onset of ovulation show ageing symptoms. The quality and developmental potential of oocyte significantly decreases with increasing time after ovulation. Ageing of oocytes display many functional changes including limited fertilization rate^2,3^, increased the frequency of ploidy anomalies^4,5^, and the malformed larvae^5–7^. For the higher vertebrates, post-ovulatory ageing of oocytes has been reported to display some other functional changes which could be observed as polyspermy, poor development of embryos and higher incidence of abnormalities in offspring probably due to aberrant epigenetic profiles [e.g.^8-10^,]. Because the oocytes contain valuable information for orchestrating embryogenesis^11^ and for remodeling the parental genomes^12^, the quality of embryos and their later developmental process are highly dependent on the oocyte integrity. In fish, oocytes display various optimum time of fertilization depending on the species and storage temperature^13^.

Once ovulation occurs, the ageing process of the oocytes is the most important factor that could affect egg quality. This has already been shown in several fish species. Maternally provided factors, including maternal mRNAs and proteins largely affect embryo development at the first steps, before zygotic transcription activates^14^. The levels and processing of maternally provided mRNAs and proteins could be impacted not only by genetic effects but also by non-genetic effects such as environmental variables like oocyte ageing^15^. Several morphological, physiological, biochemical, cellular and molecular changes occur during the progress of oocyte ageing, that results in decreasing oocyte quality^13^.

There are only few reports about the molecular changes during fish oocyte ageing^15–19^. Most reports in this field are from the studies on higher vertebrates. The specific molecular functions that determine egg quality during oocyte ageing in fish and in other vertebrates remain largely unknown. In other vertebrates, it has been suggested that increased Reactive Oxygen Species (ROS) and oxidative stress might be the initiator of oocyte ageing deteriorations [e.g.^8,20–22^,]. This leads to lowered ATP production and irregular Ca^2+^ oscillation changes. The consequences are ROS-induced mitochondrial dysfunction, ROS-induced lipid alterations and ROS-induced DNA fragmentation followed by impaired embryonic development and apoptosis^23,24^. In addition, it is believed that decreased levels of factors which are critical in the cell cycle^25^ and mitochondrial dysfunction are involved in this process^8^.

There have always been difficulties involved in the study of oocyte ageing in higher vertebrates, regarding ethical issues as well as the intrinsic nature of their reproduction biology and the difficulty of collecting adequate numbers of the oocytes. Thus, there are advantages to using fish as model animals to analyse oocyte ageing; in contrast to other animals, fish potentially have a larger number of oocytes. In addition, fish have variety modes of reproduction in oocyte ageing studies. The current study therefore, examined the possible involvement of oxidative stress in the oocyte ageing process using goldfish, *Carassius auratus*, as the model animal. The over ripening time in goldfish oocytes were characterized. Then the possible changes in the activity of antioxidant enzymes; catalase (CAT), superoxide dismutase (SOD), glutathione reductase (GR) and glutathione peroxidase (GPX), as well as TBARS as the marker of lipid oxidation during oocyte ageing were examined. Furthermore, the transcript abundance of genes involved in oxidative damage, stress response and mitochondrial function (*hsp70*, *cox1*, *sodMn, calmodulin*), genes with roles in fertilization (*vasa*), embryo development (*igf2*), cell cycling (*cyclinA, cyclinA2, cyclinB* and *jnk*) as well as the gene related to the apoptosis (*ctpb*) were investigated during oocyte ageing.

Although considerable improvements in assisted reproduction technologies are going on, there are still failures due to the oocyte ageing. There are some ethical issues that makes it difficult to study oocyte ageing on the higher vertebrates. Therefore, identifying molecular mechanisms behind the oocyte ageing process in fish and consequent declining egg quality could have important implications both for aspects of basic research as well as for the practical applications to prevent or delay oocyte ageing. The results of this study may provide a better understanding of the mechanisms involved in the oocyte ageing process which might be beneficial to other vertebrates as well.

## Results

### Embryo survival rates during *in vitro* oocyte ageing

**Figure 1:**
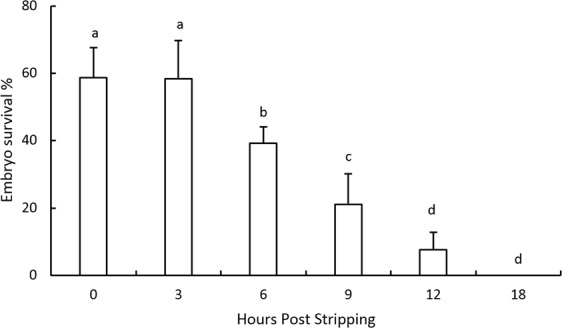
Effect of *in vitro* oocyte ageing on the embryo survival rates in goldfish (mean ± SD). Means sharing a common alphabetical symbol do not differ significantly.

The embryo survival percentages remained unchanged, at around 60%, for the eggs fertilized up to 3 hours post-stripping (Fig. 1). Then, the survival rates decreased significantly by elapsing the time storage and were 7.5% for the eggs stored *in vitro* for 12 hours post stripping (HPS). After 18 hours of oocyte storage, the fertilization rates totally lost and no viable embryos were detected.

### Relative abundance of mRNAs during *in vitro* oocyte ageing

**Figure 2:**
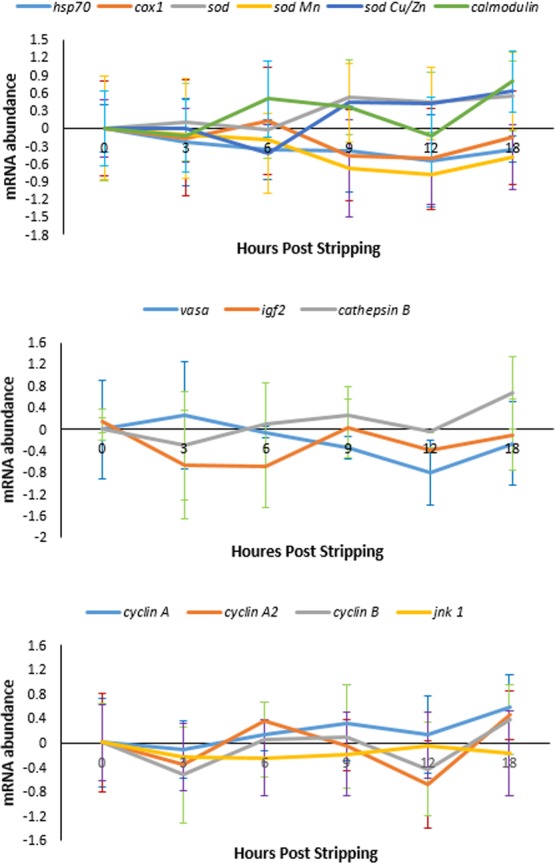
Effect of *in vitro* oocyte ageing at 20 °C on the mRNA expression levels of the selected genes in goldfish (mean ± SD).

The mRNA abundance of 13 specific transcripts were determined and compared in different aged oocytes, usibg *b-actin* as the reference gene. During 18 hours of post-stripping ova ageing, the mRNA levels in none of the evaluated transcripts exhibited significant changes (Fig. 2). Levels of *cyclinA2*, *cyclinB* and *jnk1* showed continuous down- and upregulated trends during the time interval between egg stripping and the occurrence of oocyte over-ripening.

### Activity of antioxidant enzymes during *in vitro* oocyte ageing

**Figure 3:**
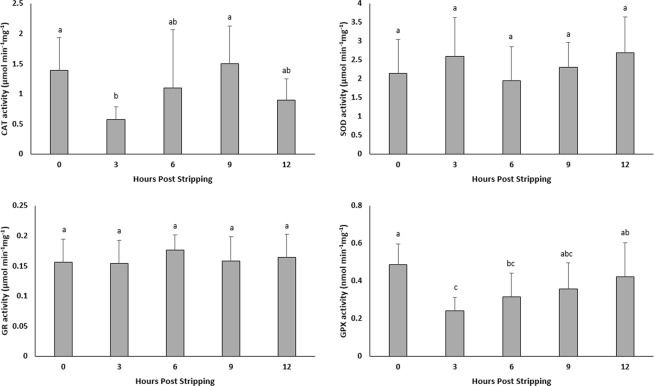
Effect of *in vitro* oocyte ageing in goldfish on the activities of catalase (CAT) (µmol/min/mg), superoxide dismutase (SOD) (µmol/min/mg), glutathione reductase (GR) (µmol/min/mg) and glutathione peroxidase (GPX) (µmol/min/mg) (mean ± SD).

The activities of SOD and GR did not change significantly during oocyte ageing (Fig. 3). The CAT and GPX activities decreased at 3 HPS and thereafter stayed almost constant with elapsing time after ovulation.

### Lipid oxidation during *in vitro* oocyte ageing

**Figure 4:**
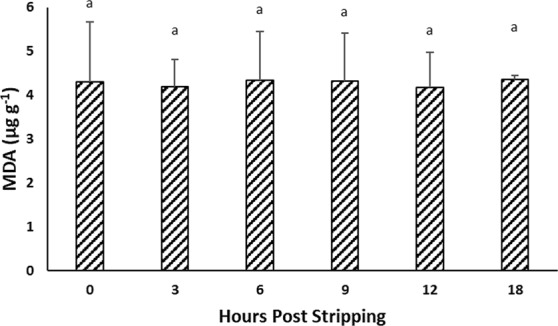
Effect of *in vitro* oocyte ageing in goldfish on TBARS, expressed as malonaldehyde (MDA) (µg g^−1^) (mean ± SD).

The level of TBARS did not change significantly and remained at around 4 µg g^−1^ malondialdehyde (MDA) during time post-stripping (Fig. 4).

## Discussion

Oocyte ageing in goldfish was associated with decreasing embryo survival rates. While the highest survival rates were obtained for the eggs fertilized up to 3 HPS, the fertilizing ability of eggs totally lost at 18 HPS. Formacion *et al*.^26^ also found that the over-ripening of goldfish eggs occurs at approximately 12 hours post ovulation with advanced degeneration by 24 hours. The time window for optimal fertilization rate is a few minutes to a few weeks. This time window highly depends on the fish species as well as the storage temperature (Reviewed by Samarin^13^).

The molecular mechanisms behind the process of oocyte ageing have not been clarified yet. Previous studies in other vertebrates have suggested that ROS and subsequently oxidative stress in oocytes increase during post-ovulatory ageing [e.g.^20–23^]. In addition, many genes used to predict egg quality of aged oocytes in other vertebrates are associated with mitochondrial function, metabolism and cell cycle control^27–30^. Microarray analysis of egg transcriptomic profiles during oocyte ageing in mice have revealed that the genes involved in oxidative stress and mitochondrial function are differentially expressed^30^. Additionally, this study suggested the alteration of expression patterns for the genes related to chromatin structure, DNA methylation, genome stability and RNA helicases during oocyte ageing. Comparatively, only a few studies have analysed egg transcriptomes during fish oocyte ageing^15,17,19^.

Our results showed no significant changes in the mRNA abundance of transcripts associated with oxidative stress (hsp70, cox and sod) during post-stripping oocyte ageing in goldfish. Esponda and Diaz^31^ showed that mRNA abundance of *hsp70* increases in aged mice oocytes. Verbeke *et al*.^32^ also found that the accumulation of damaged, oxidized and glycerated proteins might result from age-associated defects in the production of heat shock proteins. Hamatani *et al*.^30^ compared the mRNA expression profiles of mouse oocytes from young females with those of aged females. The authors reported increased *cox1 mRNA* levels in oocytes from aged mice. The mitochondrial gene *Cox*, is related to stress response that is known to catalyse the electron transfer in the respiratory chain. Therefore, mitochondrial respiratory chain in aged oocytes, might be affected by abnormal expression of *cox1* and following egg quality defects. *Sod* is an antioxidant protector in cells^33,34^ and has an important role in maintaining cellular homeostasis through removing ROS. *Sod*, an oxidative stress related gene, can protect cells from oxidative injury. Decreased mRNA levels of *sod* may decrease the capacity to cope with oxidative damage in oocytes with increasing time following ovulation. However, as we did not find such trends, we suggest that oxidative injury is not a major factor in oocyte overripening in goldfish.

Additionally, the level of TBARs which shows the extension of lipid peroxidation, did not change in the oocytes over time following stripping. The results obtained in this study showed that the activity of CAT, SOD, GR and GPX did not change significantly during oocyte ageing in goldfish, confirming that oxidative stress is not likely the main promotor in the ageing process of oocytes in goldfish. Antioxidant enzymes can reduce defective effects of oxidative stress through scavenging ROS. If oxidative stress plays critical role during the progress of oocyte ageing, then alterations in total oxidation status and antioxidant enzymes activity would be expected following ovulation. In African catfish *Clarias gariepinus*, similar results were obtained^24^, showing no involvement of oxidative stress on the oocyte ageing process at least until the complete loss of egg fertilizing ability.

The mRNA abundance of *calmodulin* in the current study showed an upward trend during oocyte ageing, although do not differ significantly. The previous studies show that increase in the ROS levels affects calcium binding in calmodulin and significantly disturb Ca^2+^ homeostasis and mitochondrial function^35,36^. A link has already been indicated between the Ca^2+^ oscillation, egg development and abnormalities suggesting that altered Ca^2+^ oscillation may lead to the poor embryo development. In other vertebrates, impaired Ca^2+^ regulation characterized by higher frequency and lower amplitude in aged oocytes [e.g.^22,37^], has been suggested as one of the molecular mechanisms behind the oocyte ageing process.

Although they do not differ significantly, the relative mRNA abundance of the transcripts related to cell cycling (*cyclinA*, *cyclinA2*, *cyclinB* and *jnk1*) were continuously decreased and increasde during the time interval between egg collection until the occurrence of oocyte ageing. Oocyte ageing in rainbow trout *Oncorhynchus mykiss* has been associated with increase in the maternal mRNA concentrations of *cyclinA*, *cyclinA2* and *jnk1*^17^. The authors concluded that since *cyclinA* regulates cell cycle progression, it is possible that post-ovulatory increase of *cyclinA* mRNA stock, participate in building the developmental competence of the egg. *jnk*, which is belonging to the family of mitogen-activated protein kinase, is an ageing-related and stress stimuli response gene. Additionally, *jnk* activity is relevant to important intracellular functions regulating cell survival and apoptosis. Previous studies in other vertebrates have suggested that critical cell cycle factors, maturation promoting factor and mitogen-activated protein kinases are decreasing during post-ovulatory ageing of oocytes^25,38,39^. The study by Xu *et al*.^38^ showed that altered levels of the factors which are critical in cell cycle and cytoplasmic changes are involved in the spontaneous activation of oocyte ageing process. In contrast, in Gilthead Sea Bream, *Sparus aurata* eggs, higher number of protein components and RNA levels involved in cell cycle regulation has been reported in higher quality eggs^40^. The observed continuous up and downward trends in mRNA abundance of the abovementioned transcripts during oocyte ageing would be interesting to be studied in the future researches.

The downward trend observed in the mRNA abundance of *vasa* in the current study during *in vitro* oocyte ageing is in accordance with the study of Hamatani *et al*.^30^ who reported that the expression of *vasa* in mouse oocytes decreases with maternal ageing^30^.

*Vasa* is a specific marker for germ cells. During embryogenesis *vasa* is expressed in the cytoplasm of primordial germ cells and has role in differentiation of the germ cells into gonads^41^, as well as a role in germ cell function^42^. Oocyte ageing in human has been suggested to be associated with a distorted secondary sex ratio in favour of males^23^. Our preliminary results with zebrafish (*vasa* transgenic strain), indicated that the number of the PGCs are significantly affected by the age of oocytes (unpublished data). Since depletion of PGCs converts the sex differentiation in favour of males in zebrafish^43^ and other fish species^44^, oocyte ageing may bias the sex ratio in favour of males or the occurrence of completely sterile individuals. This possibility should be addressed in future studies.

The results of our study indicated that the mRNA abundance of *igf2* decreased slightly up to 6 HPS and then showed an increasing trend until the 18 HPS, when no egg viabilities observed. The genes that are directly involved in the pathway of insulin growth factor did not show significant alterations during maternal oocyte ageing in mice^30^. In contrast, higher mRNA levels of *igf2* in more aged oocytes of rainbow trout was reported in comparison with the freshly ovulated ones^17^. They also reported that igf2 mRNA exhibit 2–4 fold less abundance in oocytes exhibiting low embryonic survival. The IGF axis prevents apoptosis function in the cells.

Therefore, the increased mRNA abundance of *igf2* may act as a protection against the loss of fertilizing ability and apoptosis which is the end point of the oocyte ageing process. With elapsing time after stripping, the mRNA expression of *cathepsinB* also increased. Upregulation of *cathepsinB* has been involved in cell death^45^. In African catfish *Clarias gariepinus* the mRNA abundance of an apoptosis-related gene, *cathepsinD*, demonstrated an upward trend during *in vitro* oocyte ageing^24^. Lysosomal proteases *cathepsinD* and *cathepsinB* act as stimulators of apoptosis^46^. Therefore, the observed increasing trend in the mRNA levels of *CathepsinB* was not unexpected in the current study. Our recent experiment on common carp indicated the role of apoptotic related genes on the progress of oocyte ageing^47^.

Additional analysis such as measurement of the total ROS, the indicators of mitochondrial dysfunction, egg ATP content etc., might be helpful tools to clearly understand about the possible involvement of oxidative stress in egg quality defects during oocyte ageing. Genome is transcriptionally silent from ovulation until zygotic genome activation^48^. Therefore, factors which function in post-transcriptional regulation of gene expression such as miRNA or poly(A) tail length of maternal genes may play important role during early embryo development and thus be responsible for deleterious effects of post-ovulatory ageing on the egg quality. Until zygotic genome activation, protein levels can be regulated by poly(A) tail length of maternal genes^49,50^. Delayed fertilization can affect this post-transcriptional regulation followed by developmental defects. Recently postovulatory ageing in murine MII oocytes has been associated with shortening of poly(A) tails of maternal effect genes either *in vivo* or *in vitro* culture media^51^. Poly(A) tail shortening can in turn affect the protein translation time of maternal gene transcripts followed by disturbed fertilization and developmental defects. Hence, post-transcriptional regulations and quantification of protein levels will benefit to the study on fish egg quality affected by oocyte ageing.

Epigenetics, the link between the environment and genes, has been recognized as a possible contribution to the ageing phenotype^52–54^. Some studies on higher vertebrates have already shown that post-ovulatory oocyte ageing induces epigenetic changes^9,55^. DNA methylation, histone modifications and microRNA changes are epigenetic and regulatory mechanisms that affect the gene expression without changing the original DNA sequence^56^. Factors like ageing, environment, husbandry practices *etc*. can be the cause of these epigenetic changes and developmental competence^57,58^ and they can be inherited by the offspring^59,60^. In fact, epigenetic mechanisms are the key regulators of gene transcription, with significance in responses to altered environmental signals^61^. Therefore, epigenetic modifications could be considered as a promising path for the future studies in the field of oocyte aging^1^. The results of the current study indicated that the activity of antioxidant enzymes as well as the oxidation products remains constant during oocyte ageing. Therefore, oxidative stress unlikely plays a role as an initiator or promotor in the progress of oocyte ageing in goldfish.

## Materials and Methods

### Ethics

#### Approval

The expert committee at the Institutional Animal Care and Use Committee (IACUC) of the University of South Bohemia approved the methodological protocol, experimental manipulations and sampling procedures used in this study. The permission for conducting and managing experiments involving animals was previously obtained by the co-author of this study (Certificate No. CZ 01660) according to section 15d paragraph 3 of Act No. 246/1992 Coll. Neither endangered nor protected species involved in the current study. The owner of the site; Research Institute of Aquaculture and Biodiversity of Hydrocenoses, University of South Bohemia, issued the permission for conducting the experiment.

#### Accordance

The experimental procedures were in accordance with the ethical rules of the EU-harmonized Animal Welfare Act of the Czech Republic. The unit is licensed (No. 53100/2013-MZE-17214) according to the Czech National Directive (the Law against Animal Cruelty, No. 246/1992).

### Fish

The study was conducted using 20 male and 20 female of goldfish as experimental animals. Broodfish were captured from an earthen pond at the end of spring when the average daily water temperature reached 17 °C. The captured fish were transferred to indoor cylindrical holding tanks (450 L capacity) provided with water from a recirculating system; females and males were kept separately. The fish were prepared for artificial breeding by adjusting the photoperiod to 14 L: 10 D, and the gradual increasing of water temperature to 20 °C. After 2 weeks of acclimation, the females (58 ± 11.7 g body weight, mean ± SD) and males (41.2 ± 10.6 g body weight, mean ± SD) were subjected to hormonal treatment according to Samarin *et al*.^47^. The females were checked for the occurrence of ovulation 6 hours after the second injection and this examination was repeated every 3 hours. The occurrence of ovulation was judged by applying a gentle palpation on the female abdomen. The fish was considered as an ovulated one, if the eggs could be simply stripped by applying a gentle pressure on the abdomen. The inspections were performed every 3 hours thereafter. Among the females ovulated within 3 hours, six of them, were selected arbitrary and used as the experimental fish. Fish were anaesthesized using 0.03 ml/L clove oil water bath when examining for ovulation and stripping their eggs^4^.

### *In vitro* storage of the eggs

Ova were stripped from 6 females and stored separately in sterile cell culture plates (six-well, each well diameter: 3.5 cm). The ova were stored in the laboratory incubator adjusted at 20 °C for 18 hours after stripping according to Samarin *et al*.^47^. Stored ova were fertilized at 0 (immediately after stripping), 3, 6, 9, 12 and 18 HPS.

### Artificial fertilization

In total, 20 mature males were used for the experiment. To provide a uniform fertilizing ability for all egg batches, 0.5 ml of milt was collected separately from each of three males at each HPS, pooled prior to fertilization and then used at each fertilization step. Before the egg insemination at each HPS, sperm motility was assessed separately for each male according to Fauvel *et al*.^62^. A total volume of 1.5 ml was then mixed gently and used for fertilization. At each fertilization time, ~130 eggs from each female were gently dispersed into a small petri dish and then fertilized by adding 0.15 ml of the mixed milt and 2 ml of the hatchery water. This was continued by shaking the batch for one minute. The eggs were then washed by pouring Five ml of hatchery water into each petri dish. The applied ratio of sperm to egg was previously shown be sufficient to fertilize all the eggs in the preliminary tests.

### Incubation and evaluation embryo survival rates

The eggs were washed 4–5 times with the hatchery water after the artificial fertilization, to remove the extra milt. The petri dishes were then left for 5 minutes to get assured about the attachment of the eggs to their surface. Finally, each plate was placed into a separate rectangular-shaped incubator (4.5 L capacity) supplied by recirculating water at 20 ± 0.5 °C and a flow rate of 1 L.min^−1^. The embryo survival rates were calculated 24 hours after fertilization as the percentage of live embryos to the total number of initially fertilized eggs using a stereomicroscope (Nikon SMZ745T, Japan).

### Transcriptome changes during oocyte ageing

#### RNA isolation and reverse transcription

At all HPSs the unfertilized eggs (1 g, three replicates for each fertilization time point) were snap-frozen in liquid nitrogen prior storage at −80 °C until RNA isolation.

Total RNA was isolated from 20 eggs using TRIzol reagent (Invitrogen) in combination with PureLink RNA Purification Kit (Invitrogen Corporation, Carlsbad, CA) according to the manufacturer’s instructions. The eggs were homogenized in a Precellys24 homogenizer (Bertin Instruments), 5500 rpm for 2 × 20 sec with a 5 sec interval. The RNA was treated with DNaseI to avoid contatmination of genomic DNA using a PureLink DNase Kit (Invitrogen) according to the protocol. The concentration and purtity of the RNA were assessed using a NanoDrop Spectrophotometer (ND-1000, Thermo scientific). cDNA was synthezied from 1000 ng RNA using TaqMan reverse transcription Reagents (Life Technologies) and random hexamers to prime the reaction. Reverse transcription was performed at 25 °C for 10 min, 48 °C for 1 hour and 95 °C for 5 min. Reactions without TaqMan reverse transcriptase were used as negative controls in the real-time PCR study.

#### qRT-PCR analysis

The primers were designed with Primer3 using nucleotide sequences corresponding to goldfish mRNA (NCBI) and were provided by Invitrogen^63,64^. Sequences of primers used are listed in Table 1. The relative transcript levels of 16 genes (*hsp70*, *cox1*, *sod, gpx1*, *cyclinA, cyclinB, jnkA*, *jnkB*, *caspase3A*, *caspase9*, *bax*, *bcl2, cathepsinB, cathepsinZ*, *vasa* and *igf2*) were determined by real time qPCR. The qPCR reaction was run in parallel in a LightCycler 480 (Roche), and the reaction mix consisted of 4 μl diluted (1:10) cDNA, 1 μl forward and reverse primer (final concentration of 500 nM, Table 1), and 5 μl SYBR Green-I Master (Roche Applied Science, Germany). The efficiency of all primers were evalutated using a standard curve and a melting curve of all reactions were run to control the specificity of the primers. A non-template control with water was included for each primer set. The qPCR reaction was run under the following conditions: preincubation at 95 °C for 5 min, amplification with 45 cycles at 95 °C for 15 sec and 60 °C for 1 min, melting curve at 95 °C for 5 sec and 65 °C for 1 min, and cooling at 40 °C for 10 sec.

**Table 1:** qRT-PCR primer sequences.

*Target gene*	*Forward primer* (*5*′-*3*′)	*Reverse primer* (*5*′-*3*′)	*Genbankaccession no*.
*hsp 70*	CTGTACGAGGGCATCGACTT	GCTTTCTCCACAGGCTCAAG	DQ872648
*cytochrome c oxidase I*	CTCATTGGAGGATTCGGAAA	GGAATGATGGGGGAAGAAGT	JF752338
*vasa*	CAGAGGAGGATCACGAGGAG	TTGGCCTTCATCTTTCCAAC	AY821684
*igf 2*	TGTGGAAAGGCAAACAATGA	CTAGTTCTCCACCGCAAAGG	FJ410929
*sod Mn*	TTATGCAGCTTCACCACAGC	ACACATCACCCTTTGCCAGT	JX477243
*cyclinA*	TGCATGTCTGTCCTGAGAGG	TCCACTTCCGGAGGATACAC	EU380204
*jnk 1*	GACTCCACGTTCACCGTTTT	CGTTCAAGGACATGGTCGTA	EU374209
*b-actin*	CCCTGTATGCTTCAGGTCGT	ATTGCATGGGGAAGAGCATA	AB039726
*calmodulin*	TGAAGTGGATGCTGATGGAA	TCTGATCTCCTCCTCGCTGT	JX477193
*Sod CuZn*	GTCAGACACGTCGGAGACCT	GTATTCCCCAAACAGGGTCA	JX477242
*CathepsinB*	TTCTGGAGCTCTGACGGTCT	GCCATTCACATGATGCTCAC	JX477223
*cyclinB*	AAGTTCAGGCTGCTTCAGGA	AAGCTGGAGCTGCTTCTTTG	AF273495
*sod*	GTCAGACACGTCGGAGACCT	GTATTCCCCAAACAGGGTCA	JQ776518
*cyclinA2*	TGGTGAGCTGAGCTTGATTG	CTCCTGCAATTGTGTGGTTG	AF273493

The relative gene expression was determined using the comparative CT method (2^**−∆**Ct^). To normalize relative mRNA levels, the stability of three reference genes, Glyceraldehyde 3-phosphate dehydrogenase (*gapdh*), 18S ribosomal RNA (*18s*) and beta-actin (*b-actin*), were tested. The *b-actin* was shown to be the most stable and was selected as reference gene.

### Examining the activity of antioxidant enzymes during oocyte ageing

#### Preparation of post-mitochondrial supernatant

Samples of fish eggs (approx. 400 mg) were homogenized in 0.1 M K-phosphate buffer (pH 7.4) and centrifuged at 15,000 g at 4 °C for 15 min to isolate the post-mitochondrial supernatant. Protein levels in post-mitochondrial supernatant were measured spectrophotometrically using bovine serum albumin as a standard^65^. The samples were diluted to a protein content of 10 mg/mL. All steps were carried out on ice.

#### Antioxidant enzyme activity

Catalase (CAT) activity was determined using H_2_O_2_ as a substrate to proceed reaction based on the method of Claiborne^66^. Calculations were made using a molar extinction coefficient of 40 M^−1^ cm^−1^. Total superoxide dismutase (SOD) activity was determined as formation of nitro blue tetrazolium from phenazine methosulfonate substrate based on the method of Nishikimi *et al*.^67^. Glutathione peroxidase (GPX) activity was assayed based on the rate of NADPH oxidation at 340 nm^68^ by the coupled reaction with glutathione reductase (GR), which was measured as described in Cribb *et al*.^69^.

All biochemical assays were performed spectrophotometrically in triplicate or quadruplicate using microplate reader (Tecan infinite M200, Germany). Reaction activity was linear over time. Enzyme activity was expressed as units (µmol for CAT, SOD and GR; nmol for GPX) of produced product per mg of protein per minute.

### Lipid oxydation

Lipid oxidation in oocytes was analysed by evaluation of MDA equivalents using thiobarbituric acid reactive substances (TBARS) method according to Li *et al*.^70^. Briefly, samples were incubated in darkness at room temperature (20 °C), overnight (for 15–20 hours) with thiobarbituric acid and the formed MDA was measured with a UV-visual plate reader (AF 2200: Austria) at 530 nm and expressed as µg/g.

### Statistical analysis

The normality of the data was identified using SPSS software version 18. ANOVA followed by Duncan’s multiple range test was used to evaluate the differences between the means of the groups for the examined paremeters; embryo survival rates as well as each individual gene, lipid oxidation levels and antioxidant enzyme activities. *P* < *0.05* was considered to be significant.

## Supplementary information

Supplementary Dataset 1

## Data Availability

All data generated and analyzed during this study are included in this article and its Supporting Information files in the public repository data at https://osf.io/wpfxb/quickfiles.
