# Supplementary material for: The possible involvement of oxidative stress in the oocyte ageing process in goldfish Carassius auratus (Linnaeus, 1758)
Source: Sci Rep. 2019 Jul 18;9:10469. doi: 10.1038/s41598-019-46895-1 (PMC6639355; doi:10.1038/s41598-019-46895-1)
Supplement: Supplementary file 1 — Supplementary Dataset 1 [file 41598_2019_46895_MOESM1_ESM.pdf]

Supporting information

Manuscript Title: The possible involvement of oxidative stress in the oocyte ageing process in goldfish *Carassius auratus* (Linnaeus, 1758)

Author list: Azadeh Mohagheghi Samarin, Azin Mohagheghi Samarin*, Tone-Kari Knutsdatter Østbye, Bente Ruyter, Sabine Sampels, Viktoriia Burkina, Miroslav Blecha, Tomas Policar

S1 Table. Embryo survival rates during *in vitro* oocyte ageing in goldfish.

S2 Table. mRNA abundance of the selected transcripts relative to *b-actin* during *in vitro* oocyte ageing in goldfish.

S3 Table. Activities of catalase (CAT) (µmol/min/mg), superoxide dismutase (SOD) (µmol/min/mg), glutathione reductase (GR) (µmol/min/mg) and glutathione peroxidase (GPX) (µmol/min/mg) during *in vitro* oocyte ageing in goldfish.

S4 Table. TBARS values, expressed as malonaldehyde (MDA) (µg g^{-1}) during *in vitro* oocyte ageing in goldfish.

S1

Treatment (HPS)	Replicate	Embryo survival%
1 (0)	1	61.64
1 (0)	2	60.07
1 (0)	3	63.04
1 (0)	4	46.56
1 (0)	5	72
1 (0)	6	48.8
2 (3)	1	75
2 (3)	2	56.16
2 (3)	3	68.08
2 (3)	4	40.76
2 (3)	5	57.56
2 (3)	6	52.87
3 (6)	1	32.97
3 (6)	2	46.85
3 (6)	3	41.66
3 (6)	4	40
3 (6)	5	39.61
3 (6)	6	34.16
4 (9)	1	13.84
4 (9)	2	12.55
4 (9)	3	26.6
4 (9)	4	37.32
4 (9)	5	21.53
4 (9)	6	14.28
5 (12)	1	5.05
5 (12)	2	6.5
5 (12)	3	15.32
5 (12)	4	12.8
5 (12)	5	0.99
5 (12)	6	4.46
6 (18)	1	0
6 (18)	2	0
6 (18)	3	0
6 (18)	4	0
6 (18)	5	0
6 (18)	6	0

S2

Treatment (HPS)	Replicate	hsp70	cox1	sod	sod Mn	sod Cu/Zn	calmodulin
1 (0)	1	0.050267	0.869758	-1.51327	0.860359	-1.015541	-0.476953
1 (0)	2	0.139496	0.433053	-1.02417	1.102295	-0.868605	0.06721303
1 (0)	3	0.190916	-0.26323	-1.31739	0.641912	-1.418988	-0.2959259
1 (0)	4	0.195873	0.754747	-0.9642	0.665338	-1.430562	-0.1568284
1 (0)	5	0.234278	-0.77353	2.106889	-0.38766	1.9664395	1.21774093
1 (0)	6	-0.80083	-0.99765	2.702657	-2.84068	2.8084186	-0.362354
2 (3)	1	0.70235	1.002	-0.81426	0.896061	-0.964839	-0.0941183
2 (3)	2	0.231969	0.56452	-0.69561	0.992954	-0.912946	0.20644253
2 (3)	3	-0.0817	-0.33128	-1.20199	0.464486	-1.886414	-0.1229443
2 (3)	4	-0.03885	0.355608	-1.10909	0.881358	-1.454542	-0.0702056
2 (3)	5	-0.97593	-1.43727	1.924179	-2.19951	2.0995882	-1.243279
2 (3)	6	-1.23816	-1.15931	2.502455	-1.71751	3.1415889	0.55953598
3 (6)	1	-0.21188	0.737804	-1.47521	0.37187	-1.92403	-0.5589187
3 (6)	2	0.235596	1.268157	-0.62718	0.968934	-1.741966	0.40156594
3 (6)	3	-0.138	-0.47792	-0.97029	0.34838	-1.01752	0.37696535
3 (6)	4	-0.14287	0.726087	-0.72935	0.696933	-1.213967	-0.189523
3 (6)	5	-1.14136	-1.01295	2.172121	-1.59492	1.9041845	1.2420725
3 (6)	6	-0.80096	-0.47111	1.552746	-1.92951	1.4595887	1.70762643
4 (9)	1	-0.0089	0.549967	-1.35431	0.817784	-1.283116	-0.2855578
4 (9)	2	0.18107	0.240621	-0.6675	1.348614	-0.712286	0.549242
4 (9)	3	-0.365	-0.53657	-0.74413	0.668124	-1.027776	-0.1546122
4 (9)	4	-1.75425	-1.5545	2.010123	-2.82315	2.2659505	-0.9265028
4 (9)	5	-0.02313	-0.88251	2.398496	-2.15687	2.3572274	1.91955522
4 (9)	6	-0.2598	-0.55144	1.511848	-1.94894	1.0301635	1.02606814
5 (12)	1	0.068114	0.526222	-1.36582	0.778423	-0.952477	-0.6413425
5 (12)	2	0.092824	0.399561	-0.45111	0.580338	-0.785562	0.44418771
5 (12)	3	0.061447	-0.38601	-0.80903	0.415295	-1.330605	-0.0545853
5 (12)	4	-0.90351	-1.28276	1.388776	-2.20766	1.94144	0.66918175
5 (12)	5	-0.74648	-1.58101	1.599358	-2.53851	1.5655883	-1.0757479
5 (12)	6	-1.85659	-0.77848	2.304757	-1.66134	2.1277591	-0.1283719
6 (18)	1	-0.32718	0.554807	-1.02918	0.881295	-0.504605	-0.4889294
6 (18)	2	-0.19327	0.866257	-0.64955	0.870253	-0.810647	0.19533457
6 (18)	3	-0.23186	-0.50516	-1.03043	0.372571	-1.263329	0.59457371
6 (18)	4	-0.64643	-0.53172	1.997985	-1.14715	2.0269511	1.49397953
6 (18)	5	-0.614	-1.30907	1.882733	-3.13236	2.0917379	0.90065227
6 (18)	6	-0.10279	0.000907	2.091674	-0.81821	2.2358868	2.07754658

vasa	igf2	cathepsin B	cyclin A	cyclin A2	cyclin B	jnk 1
0.112338	0.304728	-1.52474066	-0.34554	0.757859	1.346159	0.871959
0.849542	-1.056	0.08219484	-0.12861	-0.08521	0.253095	0.863895
0.848184	0.149996	-0.80318834	-0.54399	-0.01559	-0.01229	0.293512
0.275606	-1.76842	-0.29976222	-0.44556	0.297838	-0.63386	0.281938
-0.57258		1.97723948	1.406439	0.594839	0.458139	-1.30106
-1.51663	-0.13958	0.60921862	0.098419	-1.50818	-1.36988	-0.96908
1.328286	-0.64051	-1.45403898	-0.14484	0.473561	0.476861	0.027661
0.73531	-0.67961	0.03785439	-0.00795	0.405454	0.238754	0.944554
0.68617	-0.54891	-1.33061386	-0.82141	-0.26301	-0.63971	-0.37891
0.548172	-1.63443	-0.40374156	-0.32954	0.463858	-0.73284	0.077958
-1.42839		0.15038824	-0.02541	-2.49201	-1.66371	-1.11291
-0.31553	0.224825	1.20238895	0.621589	-0.71501	-0.85171	-0.92091
-0.06291	-0.07336	-2.35323047	-1.00903	0.36937	-0.06733	-0.15653
0.85483	-1.22916	-0.22616607	-0.38197	0.371434	-0.19527	0.325534
0.819555	-0.35943	-0.78672029	-0.66752	-0.22412	-0.05082	0.41998
0.580144	-2.05827	-0.36316678	-0.63397	0.399433	-0.84727	0.238533
-2.02016		2.17998449	1.714184	0.597584	0.915884	-1.75332
-0.52301	0.243244	2.11538875	1.744589	0.657989	0.556289	-0.53291
0.431666	-0.25072	-1.65231564	-0.47312	0.370284	0.473584	0.484384
1.234746	-1.08782	0.09851393	0.102714	0.301114	0.089414	0.845214
0.51509	-0.09142	-0.76697558	-0.38778	-0.05938	0.028924	0.189724
-1.24998		-0.21824953	-0.13405	-2.58565	-1.77735	-0.19655
-1.46155		2.61802743	1.897227	1.800627	2.078927	-1.43027
-1.51382	1.504848	1.43096353	0.945164	-0.07644	-0.27314	-0.98734
0.063951	-0.38799	-1.57667716	-0.51248	0.450923	0.674223	0.885023
1.068884	-1.8895	-0.12976222	-0.12056	-0.10216	-0.09386	0.306938
0.741838	-0.26751	-0.86480536	-0.54561	-0.13221	-0.18391	-0.05311
-2.05702		1.09223998	1.02644	-0.68516	-0.37686	-0.53106
-2.5894		0.41638834	0.520588	-2.42101	-1.31271	-0.36191
-2.03602	1.019325	0.83355907	0.477759	-1.16384	-1.30554	-0.47974
0.136964	0.616816	-1.09380476	-0.0096	0.808795	0.937095	0.992895
0.30207	-1.39174	-0.26484699	-0.28565	0.207753	-0.08395	0.566853
0.549353	-0.64567	-1.0575288	-0.73333	-0.18493	-0.35663	0.139171
-0.81212		1.92275107	1.366951	0.335351	0.048651	-1.38555
-1.4927		1.95753791	1.166738	0.800138	0.948438	-1.36576
-0.28652	1.000588	2.57168677	2.020887	0.754287	0.762587	0.008387

S3

Treatment (HPS)	Replicate	CAT µmol/min/mg	SOD µmol NBT/min/mg protein
1 (0)	1	0.411466133	1.096355814
1 (0)	2	1.695267195	1.826670304
1 (0)	3	1.694066673	2.526039898
1 (0)	4	1.942217083	1.29150446
1 (0)	5	1.375894916	2.613261046
1 (0)	6	1.206655513	3.508959845
2 (3)	1	0.529470142	1.29618911
2 (3)	2	0.479338726	2.307315032
2 (3)	3	0.654184177	3.067576468
2 (3)	4	0.89617	1.647713044
2 (3)	5	0.271660732	3.258612837
2 (3)	6	0.615772067	4.013679637
3 (6)	1	1.24945689	1.27425438
3 (6)	2	0.700802439	2.093289372
3 (6)	3	0.326510827	1.295533987
3 (6)	4	0.113175645	1.346783271
3 (6)	5	1.400402078	2.123965152
3 (6)	6	2.798732433	3.613390895
4 (9)	1	0.495658509	1.550694226
4 (9)	2	1.027382818	2.296127607
4 (9)	3	1.655426119	1.787359376
4 (9)	4	2.117647059	2.059464479
4 (9)	5	1.837105536	2.794596296
4 (9)	6	1.901310502	3.331823205
5 (12)	1	0.48125068	1.90532593
5 (12)	2	0.744058571	1.753017446
5 (12)	3	1.395007599	2.065957443
5 (12)	4	1.249658603	2.74775399
5 (12)	5	0.657113207	3.97589555
5 (12)	6	0.832719808	3.68960531

GR µmol/min/mg	GPx nmol/min/mg
0.120935548	0.36631535
0.164344967	0.496486902
0.112627018	0.455716517
0.211403105	0.598363714
0.187954534	0.378061391
0.141075523	0.631339857
0.151205537	0.114044434
0.192949408	0.264637986
0.136151903	0.218964906
0.208635065	0.301440866
0.112669331	0.281262726
0.124402497	0.275897554
0.155922186	0.323167463
0.195752199	0.309506641
0.196224412	0.261114603
0.19902694	0.474782421
0.175977339	0.411304763
0.137185077	0.110812182
0.146462522	0.293659251
0.225812034	0.476889771
0.109835711	0.264810332
0.184846316	0.365846966
0.13783372	0.560347699
0.144328484	0.180046798
0.145359413	0.398964751
0.211768876	0.750625594
0.141073288	0.231253932
0.215604944	0.424127136
0.1344649	0.442574972
0.136933498	0.29310405

S4

**Treatment (HPS)**	**Replicate**	**MDA (µg g-1)**
1 (0)	1	4.539081826
1 (0)	2	5.007880606
1 (0)	3	5.338477171
1 (0)	4	2.30006165
2 (3)	1	4.04709229
2 (3)	2	4.405366081
2 (3)	3	4.904474139
2 (3)	4	3.453459272
3 (6)	1	3.43994357
3 (6)	2	5.228767442
3 (6)	3	5.375611524
3 (6)	4	3.295700625
4 (9)	1	2.719702791
4 (9)	2	4.502378281
4 (9)	3	4.936112242
4 (9)	4	5.106876522
5 (12)	1	3.345348176
5 (12)	2	4.265465419
5 (12)	3	4.924423281
6 (18)	1	4.240334467
6 (18)	2	4.425549754
6 (18)	3	4.392858822
